# The Quiet Giant: Identification, Effectors, Molecular Mechanism, Physiological and Pathological Function in mRNA 5-methylcytosine Modification

**DOI:** 10.7150/ijbs.101337

**Published:** 2024-11-18

**Authors:** Ruyue Wang, Lifeng Ding, Yudong Lin, Wenqin Luo, Zhehao Xu, Weilin Li, Yi Lu, Ziwei Zhu, Zeyi Lu, Fan Li, Xudong Mao, Liqun Xia, Gonghui Li

**Affiliations:** 1 Department of Urology, Sir Run Run Shaw Hospital, Zhejiang University School of Medicine, Hangzhou, 310016, China.; 2 Department of Urology, Taizhou Hospital of Zhejiang Province affiliated to Wenzhou Medical University, Taizhou, China.

**Keywords:** 5-Methylcytosine, RNA modification, Epitranscriptomic, m5C mRNA

## Abstract

5-Methylcytosine (m5C) is a prevalent nucleotide alteration observed in transfer RNA (tRNA) and ribosomal RNA (rRNA), and it is also widely distributed in the transcriptome, serving as one of the internal modifications of messenger RNA (mRNA) in higher eukaryotes. Increasing evidence has substantiated the presence of m5C in mRNA. As research on m5C progresses, there is an initial comprehension of its molecular mechanisms and biological significance in mRNA. This work aims to provide a comprehensive summary of the most recent advancements in the identification and screening, distribution, molecular functions, and biological effects of m5C in mRNA. We outline the current status of research and provide prospects for potential future applications.

## 1. Introduction

While the exploration of m5C has been extensive within DNA, initial research into its prevalence within RNA was predominantly limited to tRNA and rRNA 1-8. Dating back to the 1970s, it was recognized that the C5 atom of cytosine could be a target for methylation in poly(A) RNA within HeLa and hamster cells 9-13. Regrettably, due to constraints in detection methodologies, m5C within the transcriptome remains an enigma for all researchers; early studies were unable to precisely validate m5C modification inside mRNA. With advancements in detection technologies, the application of bisulfite conversion coupled with sequencing has confirmed the presence of m5C modifications in the transcriptome 14-17. Later on, attention progressively shifted to discerning the impact of m5C modifications on mRNA metabolism.

**Figure 1:**
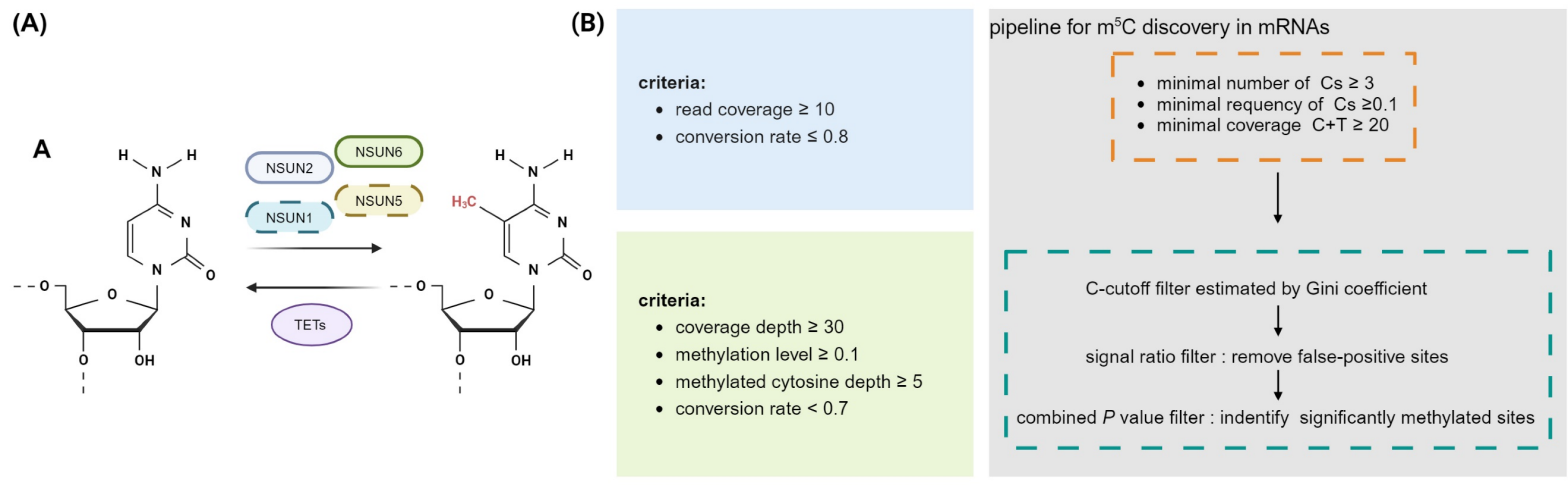
** Dynamic regulatory processes and RNA bisulfite sequencing analysis pipeline of m5C in mRNA. A,** The dynamic process of m5C and its associated regulators. NSUN1, NSUN2, NSUN5, and NSUN6 function as the catalytic enzymes of m5C methylation on mRNA, the demethylases in the TET family erase m5C methylation. **B,** Different RNA bisulfite sequencing analysis pipelines to identify m5C sites in mRNA 14,29,39. Cs: unmethylated cytosines. Image created with BioRender.com, with permission.

The dynamic and reversible methylation of RNA has opened up a frontier field in epitranscriptomics **(Fig. 1A)**. It has been confirmed that m5C has an impact on many RNA processing and biological processes. RNA m5C writers encompass members of the NOL1/NOP2/sun domain (NSUN) family, DNA methyltransferase (DNMT) family, and tRNA-specific methyltransferase (TRDMT) family 18-28. Aly/REF export factor (ALYREF) and Y-box binding protein 1 (YBX1) are currently acknowledged as two readers of mRNA m5C 29,30. Research on erasers of m5C on mRNA is limited, although some studies suggest that m5C can be erased by the ten-eleven translocation (TET) family 31. From a molecular perspective, m5C has been shown to influence a wide range of biological processes, such as cellular tolerance, migration, splicing, mRNA stability, nuclear-cytoplasmic transport, and protein translation. It also has a role in controlling stem cell reprogramming, differentiation, and development 29,30,32-40.

In order to aid researchers, this publication provides a synopsis of the present detection and identification framework, landscape, key effector molecules, and the function of mRNA m5C modification in both normal physiological and pathological conditions.

## 2. Landscape profile and identifying pipeline of m5C in mRNA

RNA cytosine can be modified by methylation on its fifth carbon atom, known as m5C modification 41. For decades, methylation of cytosine residues at position 5 in DNA has been quite familiar. Ever since it was identified in RNA in 1958, it has been reported in various RNA species, including rRNA, tRNAs, mRNAs, enhancer RNAs (eRNAs), and m5C has also been detected in long non-coding RNAs (lncRNAs), circular RNAs (circRNAs) and micro RNAs recently 42. Studies on m5C modifications initially concentrated on tRNA and rRNA, but research on mRNAs has lagged behind. However, high-throughput detection technologies for m5C modification have rapidly advanced in recent years, such as bisulfite sequencing (BS-seq), 5-azacytidine cross-linking sequence (Aza-IP) or m5C individual-nucleotide-resolution cross-linking and immunoprecipitation sequencing (miCLIP-seq) 14,43,44. Besides, a recently developed optimized sequencing method, ultrafast BS-seq (UBS-seq), based on traditional BS-seq and employing a composition of ammonium bisulfite and sulfite, has been utilized for the detection of m5C modification sites on DNA and RNA 14,45. Due to the advances of the aforementioned detection technology in high-throughput sequencing, the global distribution of m5C in cellular RNAs can be determined.

Unlike DNA, mRNA m5C modification is far less frequent, and due to the low abundance and high background noise, transcriptome-wide m5C mapping is technically challenging 39,45.

Currently, epitranscriptomic mapping can be realized via one of three strategies: fragment enrichment, sequence truncation or nucleotide transition. Discrepancies among different studies may yield distinct results; even when employing identical BS-seq protocols, the number of m5C sites remains a topic of intense debate, thus there is currently a lack of definitive unified epitranscriptomic maps of cytosine modification 46. Below, we briefly outline several m5C transcriptome maps to broadly depict the overall distribution of m5C modification on messenger RNAs.

Through utilizing RNA bisulfite conversion in combination with whole transcriptome RNA sequencing based on SOLiD technology, the first high-resolution view of cytosine modifications within the transcriptome was obtained at a single nucleotide resolution 14. Employing predefined selection criteria, 8495 novel candidate m5C sites were identified within mRNA sequences, demonstrating an enrichment of m5C within mRNA UTRs and proximal to Argonaute protein binding regions 14.

Subsequently, BS-seq has transitioned to the utilization of Illumina technology and has been employed across diverse organisms and conditions, with different studies employing diverse selection criteria for screening potential m5C candidate sites 29. Among these, one study mapped transcriptome-wide m5C profiles in human HeLa cells and multiple mouse tissues using BS-seq, identifying 5065 m5C sites on 1955 mRNAs, revealing a median methylation level of mRNA m5C sites is about 20.5%, similar to the degree of mRNA pseudouridylation. In terms of the distribution profile of these sites, m5C modifications were predominantly located within coding sequences (CDS), primarily in CG contexts, and in regions immediately downstream of the mRNA translation start site. In the m5C sequencing of mouse tissue samples, it was observed that they exhibit a median methylation level and m5C distribution pattern similar to those found in both mouse and human HeLa cells. These findings suggest a high degree of conservation in the distribution pattern of m5C within mammalian cell mRNA. Further investigations encompassed the distribution of m5C modifications in different mouse tissues and during testicular development, revealing the conservative, tissue-specific, and dynamic characteristics of m5C modifications in the mammalian transcriptome^29^. Another study focused on a comprehensive picture of cytosine methylation in the epitranscriptome of embryonic stem cells (ESCs) and the brain in total and nuclear poly(A) RNA in mice, observed a pronounced accumulation of m5C sites in the vicinity of the translational start codon (such as the end of the 5'UTR and at the very beginning of CDS), depletion in coding sequences, and mixed patterns of enrichment in the 3′ UTR. By comparing the methylation sites in ESCs and brain tissues, it was found that 57% of the methylated sites in ESCs are unmethylated in brain tissues. Moreover, these differential methylations are generally not caused by differential expression, suggesting that cytosine methylation in mRNA may occur in a highly cell- and tissue-specific manner, independent of transcript expression levels 47.

However, the estimated number of mRNA m5C sites varies greatly among different studies, with the observed results being inconsistent, making it difficult to define a universal set of mRNA substrates or common methylated target sequences **(Fig. 1B)**. Subsequently, research on m5C in mRNA has shifted towards more rigorous site identification. Rui Zhang and colleagues then develop a computational pipeline to accurately identify mRNA m5C sites, encompassing standards related to conversion efficiency, coverage and cut-off criteria **(Fig. 1B)** 39. This workflow was subsequently embraced by multiple studies 30.

**Figure 2:**
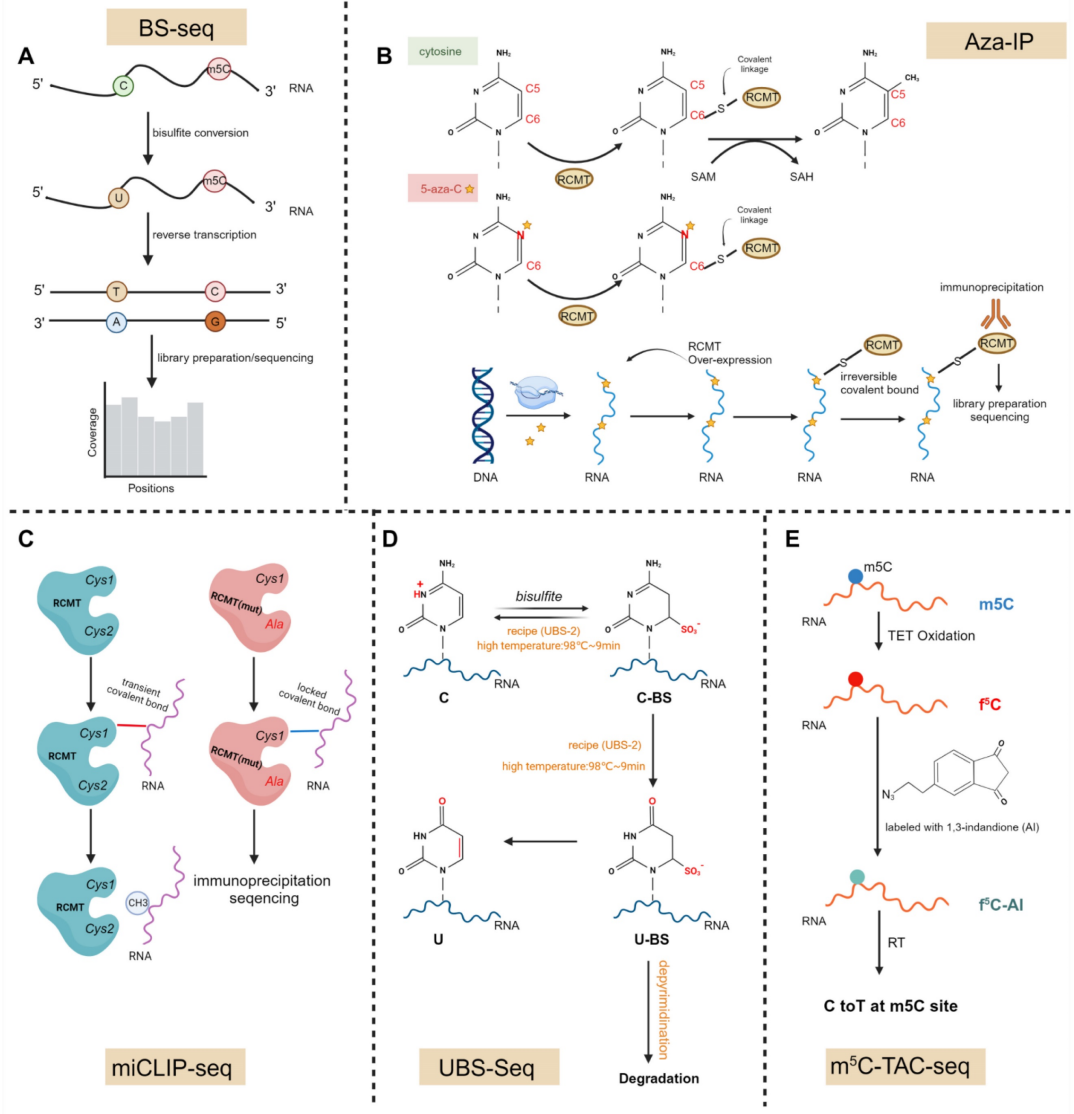
** Schematic diagram of transcriptome-wide sequencing methods for m5C. A,** In RNA BS-Seq, bisulfite treatment deaminates unmodified cytosines to uracil, while leaving methylated cytosines unchanged. **B,** 5-aza-C, a cytosine analog, is randomly incorporated by RNA polymerase into cytosine positions of nascent RNA transcripts during the transcription process. In the m5C-RCMT catalytic domain, the sulfur atom of the cysteine residue forms a covalent bond with the C6 position of the target RNA base. Subsequently, methylation of the target cytosine at the C5 position occurs through the use of the methyl donor S-adenosyl methionine (SAM). Following the methylation of C5, the covalent bond will be cleaved through subsequent beta elimination, thereby restoring the free enzyme. The covalent bond will be stabilized in the instance of 5-aza-C due to the substitution of the carbon atom at position C5 with a nitrogen atom (N), ultimately depleting endogenous enzymes in cells and leading to low methylation of RNA and DNA. **C,** The conserved cysteine residue Cys1 transiently forms a covalent bond with the methylated cytosine during the methylation process, while the second cysteine Cys2 is crucial for the decomposition of this catalytic intermediate; In miCLIP, Cys2 is mutated to alanine (Ala), facilitating the capture of the catalytic intermediate, and and permitting the methyltransferase to crosslink with its endogenous RNA target without requiring photo-crosslinking. Ultimately, these crosslinked epitope-tagged enzyme-substrate complexes. **D,** Mechanistically, two competing pathways exist in BS-seq: one that achieves the desired conversion from C to U, and another that leads to undesirable DNA/RNA degradation. UBS-seq is an ultra-fast BS sequencing method that uses a high BS concentration (∼10 M) and a high reaction temperature of 98 °C, shortening the reaction time to reduce RNA degradation and increasing the reaction temperature to achieve complete C to U conversion. **E,** In m5C-TAC-seq, m5C is oxidized to f5C, which is then labeled with an azido derivative of 1,3-indandione (AI). This process promotes the enrichment of RNA containing m5C through biotin pull-down and induces the conversion of C to T at the m5C sites. Image created with BioRender.com, with permission. (Figure D is adapted from Qing Dai *et al.*^45^, in accordance with Creative Commons Attribution 4.0 International License, http://creativecommons.org/licenses/by/4.0/).

In addition to the aforementioned methods such as BS-seq, Aza-IP, and miCLIP-seq, recently, novel sequencing technologies have also been utilized for the detection of m5C modifications on mRNA **(Fig.2A-C)**^43^,^48^-^51^. Conventional BS-seq is limited by lengthy reaction times, severe DNA/RNA damage, overestimation of the m5C level and incomplete C-to-U conversion of certain sequences. Then, novel ultrafast bisulfite sequencing (UBS-seq) is employed to detect 5-methylcytosine in DNA and RNA** (Fig.2D)**. It uses ammonium instead of sodium salt of bisulfite to achieve a much higher bisulfite concentration and a higher reaction temperature to accelerate the reaction and denature DNA and RNA 45. Consistent with the existing strategies, the detection criteria with a ≥5% unconverted cutoff, reads with more than three unconverted sites, or the number of unconverted sites accounting for more than half of the converted sites, and a binomial model was used to calculate a P value for each site. Sites with a P value less than 10^-6^ were classified as m5C sites. In addition, UBS-seq also showed that m5C sites deposited by NSUN2 but not NSUN6 are enriched in 5′-UTR regions in both HeLa and HEK293T mRNA, suggesting that m5C modification or its binding proteins may be involved in regulating mRNA translation 45.

Recently, a novel bisulfite-free method, m5C-TAC-seq, has surfaced **(Fig. 2E)** 52. This approach combines TET-assisted m5C-to-f5C oxidation with selective chemical labeling to profile m5C methylomes in human and mouse cells. Within this technique, m5C is oxidized to f5C and is subsequently labeled with an azido derivative of 1,3-indandione (AI), enabling the enrichment of m5C-containing RNAs through biotin pull-down and inducing C-to-T transitions at the m5C sites. Upon application to poly(A)-tailed RNAs in HeLa and HEK293T cell lines, m5C-TAC-seq unveiled that the majority of m5C modifications are situated in the CDS and 3′ UTR regions. Moreover, it was noted that low-stoichiometry sites are more prevalent in the 3′ UTR.

Further, some methods that were independent on the complete conversion of unmodified sites have been applied transcriptome-wide. MePMe-seq, labeling cells with a clickable metabolic precursor of S-adenosylmethionine (SAM) called propargyl-selenohomocysteine (PSH), leads to methionine adenosyl transferase (MAT)-catalyzed formation of SAM-analogue and propargylation of methyltransferase (MTase) target sites. In line with previous reports about m5C, it also indicates that m5C sites were located mainly at the end of the 5' UTR 53.

## 3. Landscape of m5C regulators in mRNA

M5C modification is a dynamic process regulated by three main molecular effectors: methyltransferases (“writers”), demethylases (“erasers”) and binding proteins (“readers”). In eukaryotes, m5C modification is catalyzed by members of the NOL1/NOP2/SUN domain (NSUN) family of proteins, NSUN1-7 and DNA methyltransferase (DNMT) homolog DNMT2 27,28,54,55 . While most of these have recognized “canonical” tRNA or rRNA targets, several have been shown to also methylate mRNA. NSUN2 was initially identified as the methyltransferase on mRNA, and then NSUN6 was shown to mediate site-specific deposition of m5C in mRNA 15,27,28,39,43. Recent research uncovered the involvement of NSUN5 in mRNA m5C deposition 52. DNMT2 (also named TRDMT1) is another type of methyltransferase at DNA damage sites 54.

**Figure 3:**
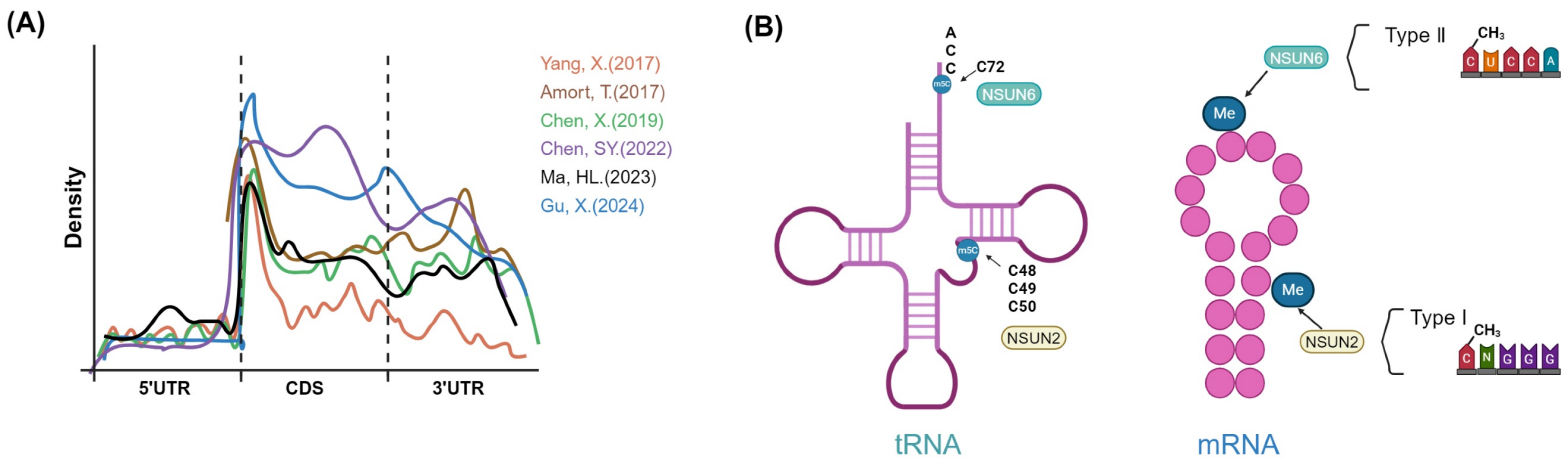
** Landscape of m5C in mRNA and m5C site preferences of NSUN2 or NSUN6. A,** Metagene plots showing the distribution of m5C sites from different studies 29,30,33,47,152,153. **B,** Schematic diagram of preferred modification sites for NSUN2 and NSUN6 on tRNA and mRNA. NSUN2 tends to methylate specific positions (C48, C49 and C50) in the vast majority of the tRNAs in humans. NSUN6 specifically targets the C72 position at the 3' end of tRNA^Thr^ and tRNA^Cys^ with a UCCA tail. NSUN2-dependent sites tend to contain *m5C*NGG motif and be located at the 5' end. NSUN6 primarily catalyzes the Type II m5C sites (*m5C*TCCA motif), and its methylated sites occur preferentially centered within the hairpin loops of stem loop structures. Image created with BioRender.com, with permission.

Overall, in mRNAs, m5C sites are distributed throughout the genome and are most frequently located in C-G rich regions, and the median methylation level of mRNA m5C sites was about 15-18% 39. The distribution of m5C sites in CDS has not yet been determined. According to Tao Huang *et al.*, m5C sites had the lowest density in CDS; this view was not supported by Xin Yang *et al.*, who indicated that m5C sites were also abundant in regions immediately downstream of translation initiation sites^29^,^39^. And according to m5C-TAC-seq, metagene analysis revealed m5C sites are evenly distributed mRNA, distinct from previous reports of 5′ UTR enrichment **(Fig. 3A)** 29,39,45,47.

Although the distribution of m5C is related to many factors such as species, cell or tissue type, developmental stage, subcellular localization, RNA structure, and sequencing methods; Overall, the median methylation level of m5C sites is about 20%. In mRNA, m5C modification sites in mRNA are predominantly situated within the CDS region, followed by the 3' UTR region, and lastly the 5' UTR. The enrichment of NSUN-dependent sites in the CDS and 3' UTR regions is similar 27,28,30,39,45,47,52,56.

NSUN2 and NSUN6 are the primary methyltransferases mediating the deposition of m5C in mRNA, and they have distinct substrate specificities. There are two types of mRNA m5C sites in animals: Type I m^5^C sites are adjacent to a downstream G-rich triplet motif and are predicted to be located at the 5^′^ end of stem-loop structures; and Type II m^5^C sites are adjacent to a downstream UCCA motif and are predicted to be located in loops of stem-loop structures 27,28,39. Moreover, Type II and Type I m5C sites had an overall similar distribution of genic locations, although compared with Type I m5C sites, a slightly lower proportion of Type II m5C sites was found in 5′UTR regions in humans and mice 28. Among which, NSUN2 is responsible for Type I mRNA m5C methylation, while NSUN6 is the methyltransferase responsible for Type II m5C sites 27,28. NSUN2 and NSUN6 tend to maintain the intrinsic substrate preferences of mRNA individually 27,28,39. NSUN2 methylates specific positions (C48, C49 and C50) in the vast majority of the tRNAs in humans and mice; these sites were located in the 5′ end of a stem region and had a 3′ G-rich triplet motif; Consistent with that, NSUN2 targeted Cs at the 5′ end of a stem region that contained a 3′ G-rich triplet motif in mRNA. NSUN2-dependent sites resemble tRNA-C49 sites most closely, and upon aligning the predicted secondary structure with the position of C49 in tRNA, it was observed that both exhibit strong predicted base pairing in the flanking regions** (Fig. 3B)** 39.

Initially, researchers found that upon knocking out NSUN2, there exists a set of m5C sites that are independent of NSUN2, and a strong 3′ TCCA motif and loop-region preference were found in these sites. Subsequently, NSUN6 was verified as the methyltransferase responsible for Type II m5C sites 27,28.

The majority of NSUN6-related miCLIP sites are located in mRNAs, in contrast to NSUN6, NSUN2-specific miCLIP sites mainly occur in tRNAs 27,28. NSUN2 and NSUN6 shared less than 4% of the identified miCLIP targets; NSUN2 preferred 5′ UTR, while NSUN6 mostly methylated 3′ UTRs. Type II and Type I m5C sites had an overall similar distribution of genic locations, although compared with Type I m5C sites, a slightly lower proportion of Type II m5C sites was found in 5'UTR regions in humans and mice, it is consistent with NSUN6 predominantly targeting the 3'UTR. Moreover, BS-seq of nuclear and cytoplasmic fractions suggests that NSUN2/Type I sites are made early on during nuclear mRNA processing, while NSUN6/Type II sites are formed on mature mRNA in the cytoplasm. Type I m5C sites were mainly enriched in the nuclear fractions (nucleus, nucleolus, lamina and nuclear pore) 28. Transcripts containing Type II m5C sites were mostly enriched near the endoplasmic reticulum membrane (ERM) and outer mitochondrial membrane (OMM), but not in the endoplasmic reticulum lumen. This is consistent with the fact that genes encoding molecules with mitochondrial and transport functions are enriched with m5C-containing genes in mouse muscle and heart 39. NSUN2 and NSUN6 have different intracellular localization, NSUN2 is located in both the nucleus and cytoplasm, while NSUN6 is mainly located in the cytoplasm; however, this is not absolute, as the distribution of different intracellular enzymes may vary 28.

Currently, it is widely acknowledged that NSUN2 and NSUN6 are the two major enzymes mediating m5C modification in mRNA, but which one predominates in cells remains elusive.

Some studies suggest the majority of sites in both mRNAs and noncoding RNA were dependent on NSUN2, but this finding is not entirely universal. m5C sites in mRNA from the HEK293T cells exhibited twofold to threefold higher enrichment as type II sites compared with those from HeLa cells 45, and NSUN6 knockout HeLa cells showed a milder impact on proliferation defect compared to HEK293T, which may be because NSUN6 contributed little to mRNA m5C in HeLa cells 28. Hence, variations in the expression of methyltransferases may contribute to the discrepancies observed in m5C methylation patterns across different cell lines, this could be related to methylation abundances, genic locations and proportions, and targeted genes in different cells. Further exploration into the m5C regulators is warranted.

Individual m5C sites have rapid evolution and weak cross-species conservation. At the gene level, the overall methylation levels of individual genes were not conserved between species, the difference may be attributed to motifs and structures 56,57. The conserved m5C sites shared a strong stem-loop, and the sites with loss of methylation between species had weak stem-loop. However, the conserved type II sites showed a less stringent stem-loop structure requirement, which indicated that human NSUN6 had more relaxing structure requirement to broaden its methylation target selection 56,57.

Recent studies have provided some intriguing insights: in addition to Type I and Type II sites, two more clusters (termed as Type III and IV) appeared, Type III sites (found in both early brain developmental stages in vertebrates and Nocodazole-treated HeLa cells) and Type IV sites (only found in Nocodazole-treated HeLa cells) 58. The sequence motifs of Type III and IV sites resembled C3782 and C4447 in human 28S rRNA methylated by Rcm1 (NSUN5) and Nop2 (NSUN1), respectively 58-60. New evidence also supports the catalytic function of NSUN5 in mRNA m5C modification and confirms that NSUN5-dependent locations have an abundance of the GCm5CANATG motif 52. Moreover, previous studies have also indicated that NSUN5 can regulate mRNA m5C modification in maternal-to-zygotic transition or carcinoma 35,61,62. Taken together, these findings indicate that NSUN5 and Nop2 are potential new mRNA m5C writers. Beyond that, there is some evidence for the modification of at least specific mRNAs by NSUN4 63, NSUN7 64, and TRDMT1 65.

## 4. Molecular Function of m5C in mRNAs

### 4.1 Stability

**Figure 4:**
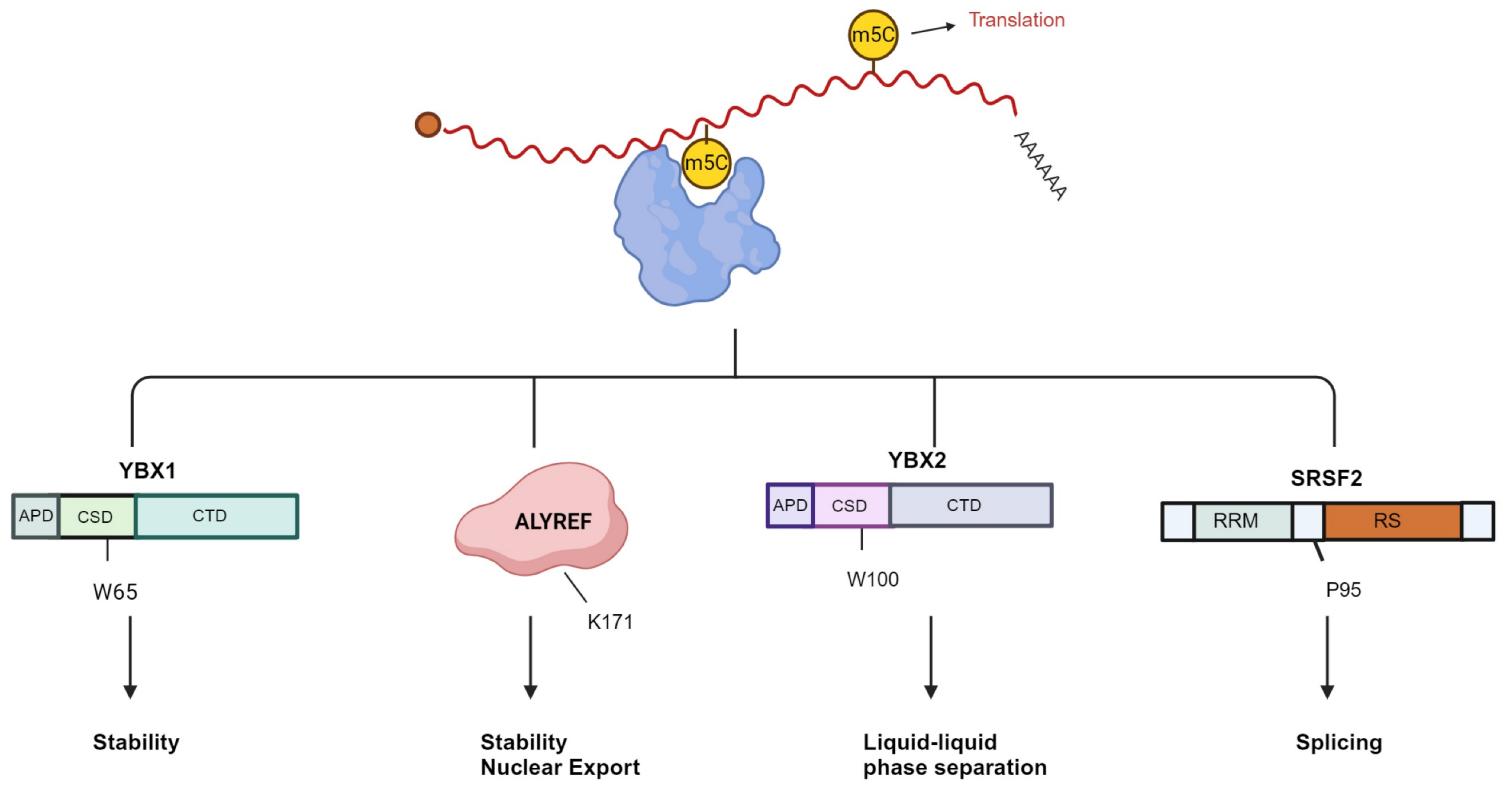
** Overview of the recognition proteins related to m5C of mRNA.** The mRNA m5C contributes significantly to translational control. YBX1 enhances mRNA stability, and W65 in YBX1 CSD is the key residue that recognizes the m5C nucleotide. ALYREF recognizes m5C modification to promote mRNA export; the K171 residue binds to the m5C-containing oligonucleotide. RNA m5C enhances liquid-liquid phase separation of YBX2, and W100 is the key residue that recognizes m5C. SRSF2 binds the m5C mark and regulates alternative splicing effects of NSUN2-mediated m5C through its reader function. The P95H mutation reduces the binding affinity of SRSF2 for RNA m5C. Image created with BioRender.com, with permission.

The primary method of identifying m5C molecular function is to identify and further study RNA binding proteins that prefer to combine methylated mRNA sequences. YBX1 and ALYREF are currently known as mRNA m5c readers 30,32. Additionally, the YBX2, serine/arginine-rich splicing factor 2 (SRSF2), fragile X mental retardation protein (FMRP), RAD52, and Lin-28 homogen B (LIN28B) genes are also likely to have reader traits **(Fig. 4)** 33,66-69.

**Figure 5:**
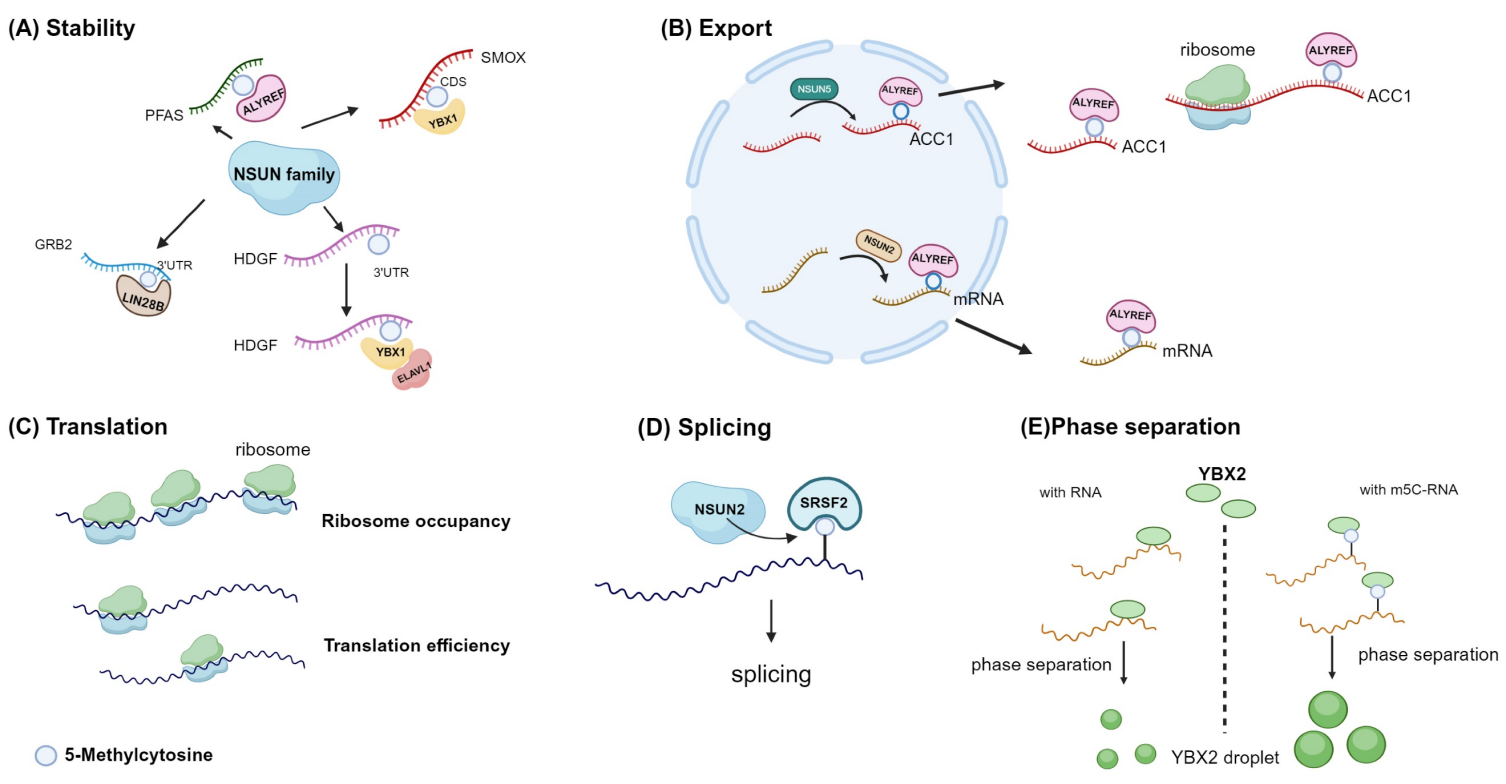
** Molecular function of m5C in mRNAs.** Diverse biological functions of m5C methylation in mRNA across different studies. **A,** m5C promotes mRNA stability.** B,** ALYREF mediates nuclear export.** C,** m5C could regulate translation efficiency and ribosome occupancy through an unknown intermediary.** D,** SRSF2 preferentially binds m5C-mRNA. NSUN2 loss reduces mRNA m5C levels and alters SRSF2 RNA binding and splicing. **E,** RNA 5-methylcytosine regulates YBX2-dependent liquid-liquid phase separation. Image created with BioRender.com, with permission.

Currently, the research on the impact of m5C modification on mRNA stability is extensive, with multiple studies demonstrating its capacity to enhance mRNA stability **(Fig. 5A)**. A strong correlation exists between the stability of mRNA and m5C modification at the transcriptional level. In endometrial cancer cells, NSUN2 confers resistance to ferroptosis by modulating the m5C modification of SLC7A11 mRNA and boosting its YBX1-dependent mRNA stability 70. Similarly, NSUN2 promotes the stability of fatty acid-binding protein 5 (FABP5) mRNA by m5C methylation 71. ALYREF contributes to the NSUN2/PFAS oncogenic cascade by enhancing the stability of PFAS mRNA during retinoblastoma progression 72. YBX1 binding to the m5C site in the coding sequence region of the SMOX transcript increases stability in esophageal squamous cell carcinoma 36. In non-small cell lung cancer, NSUN2 enhances the stability of NRF2 mRNA by depositing m5C modification in the 5' untranslated region 73. YBX1 also recruits ELAV-like 1 (ELAVL1) and poly(A)-binding protein, cytoplasmic 1a (PABPC1a), which in turn enhance mRNA stability 30. In contrast, NSUN2 preferentially catalyzes m5C methylation of IRF3 mRNA and enhances its degradation, hence contributing to the function of antiviral innate immunity 74.

To summarize, it can be inferred that m5C modification has an impact on mRNA stability, regardless of whether it is deposited in the 3' untranslated region, 5' untranslated region, or coding sequence area, mostly leading to an increase in mRNA stability. Furthermore, both ALYREF and YBX1 have the ability to identify and attach to m5C modifications, thereby impacting the stability of mRNA.

### 4.2 Export

ALYREF can specifically identify m5C, which leads to the promotion of selective mRNA export 29. ALYREF specifically recognizes and binds to the 3' untranslated region of ACC1 mRNA, which is mediated by NSUN5 in an m5C-dependent manner, promoting its nuclear export 62. In a similar vein, when NSUN2 is knockout, it hinders ALYREF's ability to identify CDKN1A mRNA, leading to reduced nuclear-cytoplasmic shuttling of CDKN1A mRNA, subsequently resulting in decreased CDKN1A translation, accelerating the cell cycle, and promoting lipid generation **(Fig. 5B)** 75. ALYREF recognizes YBX2 and SMO mRNA, which have m5C modification, and transports them from the nucleus to the cytoplasm. This process results in the upregulation of YBX2 and SMO proteins, which in turn restrict lipid production and promote muscle generation 32.

### 4.3 Translation

It influences translation in two ways: first, as previously indicated, by affecting the nuclear-cytoplasmic distribution of mRNA, and second, by regulating translation efficiency **(Fig. 5C)**. However, the translational implications of m5C remain ambiguous, and the intermediary involved remains unknown. Polysome profiling study indicates a negative correlation between m5C and translational status 39,40. m5C sites in the CDS region can negatively regulate *in vivo* translation, a phenomenon not observed in genes with m5C sites in the 5' UTR or 3' UTR 39. Additionally, certain studies suggest that this pattern is particularly noticeable in CDS sites, yet observable in 5' UTR sites, while 3' UTR and intronic sites do not display a distinct pattern 40. NSUN6 methylation is involved in the regulation of translation termination, and it is possible that NSUN6-dependent methylation is a component of the quality control mechanism that ensures translation termination 27. Nevertheless, research has also noted that there is no disparity in the effectiveness of translating NSUN6-modified mRNA between normal WT cells and NSUN6 KO in HAP1 cells 57. NSUN2 methylation of the p27 5' UTR inhibits p27 translation 76. NSUN2 and METTL3 cooperatively modify the p21 mRNA 3' UTR region, enhancing p21 expression at the translational level 77. NSUN4 facilitates m5C deposition in 3' UTR of mesenchymal stem cells. This, together with METTL3-mediated m6A, in conjunction with METTL3-mediated m6A, synergistically boosts the translation of SRY-box transcription factor 9 (SOX9) mRNA 38.

### 4.4 Others

Other than the two classical m5C reader proteins already stated, novel m5C binding proteins have been discovered through multiple studies. Serine/arginine rich splicing factor 2 (SRSF2) has also been identified as a reader of m5C, suggesting that m5C may regulate alternative splicing by recruiting SRSF2 **(Fig. 5D)** 33. The Y-box binding protein 1 (YBX1) has a strong affinity for m5C-modified mRNA, specifically through its cold shock domain 30. Similarly, YBX2 has recently been identified as a novel m5C binding protein, wherein W100 (W101 in mice) is the key residue for recognizing m5C. The m5C-modified RNA has a high affinity for YBX2, facilitating the phase separation of YBX2 **(Fig. 5E)** 66.

## 5. Biological function and disease phenotype

### 5.1 Embryonic development

During development in both vertebrate and invertebrate species, the number of m5C sites dropped dramatically after the maternal-to-zygotic transition (MZT) and remained low throughout the rest of the developmental stages. Along with the development of more organized 5' end regions in mammals came the acquisition of m5C sites at the 5' end of mRNAs, mediated by NSUN2. Moreover, through evolution, humans have specifically expanded the methylation target selection of maternal mRNA regulation by NSUN6, acquiring thousands of Type II m5C sites 56. m5C preserves mRNA stability across the maternal-to-zygotic transition in zebrafish. Early embryonic abnormalities in gastrulation occur as a result of mutations in the m5C recognition site YBX1 37. In Drosophila, the YBX1 homologue, Ypsilon schachtel (Yps), facilitates germ-line stem cell maintenance, homeostasis, proliferation and differentiation through binding to m5C-containing RNAs 78. During oogenesis and ovarian aging, NSUN5 alters alternative splicing patterns in the CDS region. It regulates mRNA decay and stability across multiple stages of the maternal-to-zygotic transition 35. Collectively, various pieces of evidence suggest that m5C plays a pivotal role in early development.

### 5.2 Metabolism and inflammation

A heart necroptosis-associated piRNA (HNEAP) interacts with DNMT1 and attenuates m5C methylation, regulating cardiac injury caused by necroptosis in ischemic heart disease 79. NSUN2 could activate the antioxidant stress response and inhibit DOX-induced myocardial injury through m5C methylation modification 80. NSUN5 promotes m5C methylation against ferroptosis in acute-on-chronic liver failure 81. YBX1 can regulate autophagy and adipogenesis at the post-transcriptional level via the m5C pathway 82. NSUN2 and ALYREF control adipogenesis by controlling cell cycle progression 83. Apart from impacting lipid metabolism, m5C modification also influences glucose metabolism. NSUN2 is a direct glucose sensor, activated by glucose to suppress cGAS/STING pathway 84. NSUN2 also regulates metabolism recoding through other mechanisms, whereas other m5C regulators, such as TET3 and NOP2, also influence metabolic plasticity via m5C modification 85-87.

### 5.3 Immunity

Many studies conducting integrative multi-omics analyses from public databases suggest that RNA m5C modification or its regulators are associated with immune infiltration and the tumor microenvironment, holding the potential application value for predicting disease prognosis and immunotherapy 88-92. Bioinformatics analysis m5C-related regulators may have the potential to serve as gene signature for prognostic prediction and clinical assessment in tumor immunity, such as in colon adenocarcinoma, prostate cancer and cervical cancer 88-96. NSUN2 is coupled with RoRγt to co-transcriptionally catalyze specific cytokine mRNAs, thus shaping the fate of Th17 cells and promoting colitis 97. Moreover, NSUN2 is involved in vascular endothelial inflammation 98,99. The m5C level in CD4+ T cells of patients with systemic lupus erythematosus (SLE) was lower compared with that in healthy controls 100. NSUN5/TET2-mediated chromatin-associated RNA modifications convert 5-methylcytosine to 5-hydroxymethylcytosine, regulating glioma immune evasion 61. Besides, Luo *et al.* combined bioinformatics and experiment validated that NSUN6 coordinated bladder cancer progression via macrophage reprogramming 101.

### 5.4 Viral infections

Endogenous NSUN2 levels decrease during SARS-CoV-2 and other various viral infections to boost antiviral responses for effective elimination of viruses 74. Besides, reduced NSUN2 expression is sufficient for maintaining HBV m5C to facilitate efficient viral infection 102. m5C is a post-transcriptional regulator of both splicing and function of HIV-1 mRNA, loss of m5C interferes with alternative splicing of HIV-1 mRNAs 103.

### 5.5 Genetic disease

Numerous indications underscore the association of m5C with neurodevelopmental disorders. NSUN6 and NSUN7 exhibit differential expression in Alzheimer's disease and traumatic brain injury 104. NSUN6 might regulate through fine-tuning of their expression. Biallelic variants in NSUN6 cause an autosomal recessive neurodevelopmental disorder 105. NSUN2 is also linked to an intellectual disability associated with a spectrum of ocular symptoms, brain anomalies, chronic nephritis, and Dubowitz syndrome 106-114. Whereas NSUN5 and its two truncated paralogs map to the commonly deleted region or its flanking sequences in Williams-Beuren syndrome, potentially contributing to specific phenotypic characteristics 115.

### 5.6 Cancer

Multiple mRNA m5C effectors, which regulate m5C modifications, have been identified as participants in cancer development and progression. Specifically, m5C modifications contribute to multi-tumors by regulating mRNA stability, splicing, expression, and translation. M5C regulates cell proliferation, metastasis, tumorigenesis, differentiation, resistance, ferroptosis and microenvironment in various types of carcinoma, such as bladder cancer, esophageal carcinoma, colorectal cancer glioma and so on 30,33,34,36,62,68,70,116-126. Beyond the aforementioned modalities, m5C modification on mRNA involve in metabolism, including glucose metabolism, lipid metabolism, amino acid metabolism 62,84-87,127-130. Apart from this, m5C can exert effects on stem cell homing and self-renewal in certain tumor progression 131. That is, m5C on mRNAs participates extensively in tumorigenesis and tumor progression.

Here, we only partially delineate the role of m5C modification on mRNA. For a more comprehensive and in-depth understanding of m5C modification, readers are encouraged to refer to recent comprehensive review articles 132-143.

## 6. Challenges

m5C levels are 3-10-fold rarer than m6A, at levels ranging from 0.03-0.1% of cytosines 144,145. For a long time, the precise mapping of m5C has been a challenging enigma. The two main methods currently for study m5C modification are liquid chromatography-mass spectrometry (LC-MS) and high-throughput sequencing approaches.

LC-MS has limitations in measuring mRNA modifications, as it can estimate the overall level of modifications in a sample but cannot assign these modifications to specific sites. Additionally, compared to tRNA and rRNA, the relative levels of modifications in mRNA are low, making it difficult to completely avoid low-level contamination from highly expressed tRNA and rRNA, which can lead to inaccurate results^146^. Over the past decade, high-throughput sequencing has been pivotal in driving advancements in the exploration of m5C modifications in mRNA.

However, sequencing-based methods also face a variety of issues, the most prominent of which is the interference of false positive background noise, which may originate from biological, library preparation, and data analysis sources. An increasing number of sequencing technologies have emerged for identifying m5C modifications in the transcriptome, with BS-SEQ remaining one of the most mainstream and widely used techniques. Traditional BS-SEQ faces several challenges in terms of accuracy and sensitivity: incomplete and uneven conversion in structured regions can introduce false positives; RNA degradation hinders the detection of low abundance and low-input samples; C to U conversion reduces sequence complexity, leading to library construction and mapping issues; and differential data analysis can result in identification discrepancies^52^. In summary, more precise and sensitive detection technologies are still required to achieve comprehensive and accurate detection of m5C modifications.

On the other hand, research on the diagnostic and clinical applications of m5C modifications in disease is currently limited. Exploration of its implications in disease diagnosis, prognostic analysis, and treatment efficacy is also in its early stages, primarily revolving around studies of enzymes related to m5C modification or the mechanisms resulting from changes in m5C modification of certain RNAs. However, these investigations are relatively indirect or constrained, lacking large-scale clinical sample sequencing to further directly and extensively explore the application of m5C modification in disease diagnosis. Additionally, there is a need for more cost-effective and widely applicable sequencing technologies to achieve absolute quantification and broaden their suitability for low-input or single-cell samples.

## 7. Future perspective

RNA bisulfite sequencing, a groundbreaking method established in 2012, has set a new standard for single-base resolution m5C identification. Despite its prominence, traditional BS-seq methods exhibit limitations that necessitate refinement. These methods rely on the indirect characterization of m5C sites through Cs to U conversion, a process demanding prolonged reactions and harsh conditions, including high temperatures. Such conditions not only risk RNA damage but also lead to potential overestimation of 5mC levels, introducing bias in fragmenting at C sites. Incomplete conversion within structured regions can lead to false positive signals in m5C detection. However, in the effort to avoid such false positives, there is a considerable risk of losing a substantial number of m5C sites during the analysis. Furthermore, stringent reaction conditions result in significant RNA degradation, posing a barrier to m5C detection in low-input samples and low-abundance RNA. Lastly, the complete conversion of all Cs to Us significantly reduces sequence complexity, thereby impeding the detection of m5C in low-complexity RNA sequences, such as chromatin-associated RNA (caRNA). Consequently, inconsistencies arise, particularly when detecting low-abundance RNAs such as mRNA. Varied studies have reported significant disparities in the number and location of modified sites, underscoring the need for more sensitive and robust methodologies to identify and quantify genuine m5C sites in mRNA. Presently, innovative approaches such as Ultrafast bisulfite sequencing, MePMe-seq, Aza-IP, miCILP, Tet-assisted peroxotungstate oxidation sequencing (TAWO-seq), m5C-TAC-seq, and nanopore direct RNA sequencing are emerging as promising avenues for m5C modification detection 43,45,52,147,148.

Further investigation into the molecular function of m5C is required. Its developmental dynamics, function, and evolutionary implications in mRNA remain largely enigmatic, particularly in cross-species research. Despite numerous studies suggesting m5C modification's impact on mRNA translation, the precise underlying mechanism remains elusive.

M5C is intricately involved in various physiological and pathological processes, elevating the significance of research into its pharmacological aspects. Noteworthy studies include Robert A. Zimmermann *et al.*'s application of distinct chemical space docking screening strategies to identify ligands for the low predicted druggability RNA methyltransferases DNMT2 and NSUN6 149. Additionally, Yongfeng Tao *et al.* employed cysteine-directed activity-based protein profiling (ABPP) to uncover azetidine acrylamides that act as stereoselective covalent inhibitors of human NSUN2 150. Recently, Baoxiang Chen *et al.* utilized virtual screening based on molecular docking to identify small molecule inhibitors of NSUN2 from the ChemDIV database, offering a novel and promising therapeutic option for colorectal cancer immunotherapy 87. In recent times, a novel approach has emerged based on CRISPR-Cas13d, termed the reengineered m5C modification system (referred to as "RCMS"). This method involves the fusion of components of m5C methyltransferases (NSUN2/NSUN6) or demethylases (Tet2 catalytic domain (CD)) into dCasRx, thereby enabling the precise manipulation of methylation events 151.

As previously mentioned, the biological significance and molecular functions of m5C require further exploration. It remains unknown in which biological contexts its levels fluctuate significantly. Ultimately, m5C modification remains an enigma for us, awaiting further unraveling of its mysterious veil.

## Abbreviations

m5C: 5-Methylcytosine
tRNA: transfer RNA
rRNA: ribosomal RNA
mRNA: messenger RNA
DNA: DeoxyriboNucleic Acid
NSUN family: NOL1/NOP2/sun domain family
DNMT family: DNA methyltransferase family
TRDMT family: tRNA-specific methyltransferase family
ALYREF: Aly/REF export factor
YBX1: Y-box binding protein 1
TET: ten-eleven translocation
eRNAs: enhancer RNAs
lncRNAs: long non-coding RNAs
circRNAs: circular RNAs
BS-seq: bisulfite sequencing
Aza-IP: 5-azacytidine cross-linking sequence
miCLIP-seq: individual-nucleotide-resolution cross-linking and immunoprecipitation sequencing
UBS-seq: ultrafast BS-seq
CDS: coding sequences
UTR: Untranslated Region
ESCs: embryonic stem cells
m5C-TAC-seq: m5C-TET-assisted chemical labeling sequencing
SAM: S-adenosylmethionine
PSH: propargyl-selenohomocysteine
MTase: methyltransferase
ERM: endoplasmic reticulum membrane
OMM: outer mitochondrial membrane
SRSF2: serine/arginine-rich splicing factor 2
FMRP: fragile X mental retardation protein
LIN28B: Lin-28 homogen B
SLC7A11: solute carrier family 7 member 11
FABP5: fatty acid-binding protein 5
PFAS: phosphoribosylformylglycinamidine synthase
SMOX: spermine oxidase
NRF2: nuclear factor erythroid 2-related factor 2
IRF3: Interferon regulatory factor 3
ELAVL1: ELAV-like 1
PABPC1a: poly(A) binding protein, cytoplasmic 1a
ACC1: acetyl-CoA carboxylase ACC1
CDKN1A: cyclin dependent kinase inhibitor 1A
METTL3: methyltransferase 3
SOX9: SRY-box transcription factor 9
MZT: maternal-to-zygotic transition
Yps: Ypsilon schachtel
piRNA: PIWI interacting RNA
caRNA: chromatin-associated RNA
TAWO-seq: Tet-assisted peroxotungstate oxidation sequencing
ABPP: activity-based protein profiling
LC-MS: liquid chromatography-mass spectrometry
